# Gene expression as a biological dosimeter: effects of different neutron energies

**DOI:** 10.1007/s00411-026-01237-4

**Published:** 2026-07-13

**Authors:** Rashmi Basavaraj, Adam Vanasse, Constantinos G. Broustas, Guy Garty, Naresh T. Deoli, Andrew D. Harken, Sangho Nam, Cameron Goodwin, Christopher Hemme, Sally A. Amundson, Michael Antosh

**Affiliations:** 1 Physics Department, University of Rhode Island, Kingston, RI USA; 2 Present Address: Department of Physics, Boston College, Boston, MA USA; 3 Center for Radiological Research, Columbia University Irving Medical Center, New York, NY USA; 4 Radiological Research Accelerator Facility, Columbia University, Irvington, NY USA; 5 Rhode Island Nuclear Science Center, Narragansett, RI USA; 6 Department of Mechanical, Industrial and Systems Engineering, University of Rhode Island, Kingston, RI USA; 7 Present Address: Project Omega Co, Newport, RI, USA; 8 Biomedical and Pharmaceutical Sciences Department, University of Rhode Island, Kingston, RI USA

**Keywords:** Radiation dosimetry, Gene expression, Neutron radiation, Neutron energy, Nuclear reactor

## Abstract

In the radiation biodosimetry field, transcriptomic studies seek to determine whether the expression of a small set of genes can be used to estimate the radiation dose that a person was exposed to. Most such studies consider only photon exposure, but in the detonation of an improvised nuclear device, neutrons would likely comprise a significant proportion of the dose. In this study, we compare the effects on gene expression in human blood between two neutron-producing radiation sources: a source designed to mimic the neutron energy spectrum of a nuclear weapon (CINF; the Columbia IND Neutron Facility at the Radiological Research Accelerator Facility) and a source from a nuclear reactor (RINSC; the Rhode Island Nuclear Science Center). The radiation sources have very different neutron energy spectra: mainly fast neutrons (CINF) versus thermal neutrons (RINSC). We have compared differential gene expression at 24 h after exposure to 0, 0.5, 1, 2, and 4 Gy total doses delivered to human blood samples from the same donors in parallel at the two sites. Our analysis suggests that there is a core response to radiation that is conserved at both sites, with larger magnitude and additional components in the CINF response. These results suggest that neutron energy does affect gene expression, and that this must be accounted for in the development of biodosimetry approaches.

**Supplementary Information:**

The online version contains supplementary material available at 10.1007/s00411-026-01237-4.

## Introduction

The effort to build a radiation dosimeter based on gene expression is motivated by the belief that it would be a very important tool in the event of a situation where a large population is irradiated. These situations may well include the use of a nuclear weapon or improvised nuclear device (IND), or a catastrophic event involving a nuclear power plant (Amundson 2023). Detonation of a nuclear device or a reactor disaster would likely result in a neutron component of the exposures (Stricklin et al. 2018). In a situation such as these, knowledge of the radiation dose could help to guide treatment, and it could also provide knowledge about the future cancer risk (Hall and Giaccia 2012; Satyamitra et al. 2023). Although neutrons generally have a greater biological effectiveness than photons, previous work in radiation biodosimetry has focused largely on photons. A number of studies have used neutron radiation, however, and include studies of cytogenetic and protein biodosimetry endpoints after exposure to TRIGA reactor neutrons (Prasanna et al. 1997; Ossetrova et al. 2018) or to a neutron beam produced by 66 MeV protons impinging on a Beryllium target (Vandersickel et al. 2014).

With increasing interest in developing biodosimetry and other medical countermeasures directed towards a nuclear detonation, the Columbia University Radiological Research Accelerator Facility (RARAF) has developed an irradiator that produces a neutron spectrum based on that 1.5 km from the hypocenter at Hiroshima (Xu et al. 2015). The Columbia IND Neutron Facility (CINF) has been used to study neutron effects on cytogenetic (Pujol-Canadell et al. 2020; Shuryak et al. 2021), metabolomic (Laiakis et al. 2017, 2019), miRNA (Yadav et al. 2020) and mRNA (Broustas et al. 2017a, b, 2018) biodosimetry. Gene expression analysis in human blood exposed to neutrons and X-rays revealed 125 differentially expressed genes and 112 enriched gene ontology terms. The TP53 signaling pathway and DNA damage response were significantly affected by all radiation doses, except for the lowest X-ray dose of 0.1 Gy (Broustas et al. 2017a). Comparing the effects of neutron and X-ray exposure in mice, both radiation types resulted in a large number of differentially expressed genes. However, neutron exposure predominantly downregulated genes, while X-ray exposure led to both upregulation and downregulation at all doses and time points (Broustas et al. 2017b). Mice exposed to neutron, photon, or mixed radiation showed a higher number of upregulated genes at roughly equitoxic doses of X-rays (3 Gy) or neutrons (0.75 Gy) (Broustas et al. 2018). Analysis revealed enrichment of protein ubiquitination pathways across all radiation types and doses. Neutron exposure specifically downregulated genes involved in transfer RNA processes and upregulated eukaryotic initiation factor 2 (EIF2) (Broustas et al. 2018).

Furthering the work in this field, this paper examines the difference in gene expression response between two different radiation sources that both produce neutrons and photons: CINF, an accelerator based source optimized to mimic IND exposures (Xu et al. 2015) and RINSC, a 2 MW research and training reactor. As detailed in the discussion, the difference between neutron energies at the two sites is expected to result in a 5.3-fold difference in the radiation protection quantity equivalent dose (ICRP 2007).

## Materials and methods

### Collection of samples

Ten blood samples of 2.7 mL each were collected from each donor under IRB protocol number IRB-AAAS5693 at Columbia University. There were 12 donors total, 6 male and 6 female. Blood was drawn into vacutainer tubes containing the anticoagulant sodium citrate. No personally identifying donor information (name, address, etc.) was associated with the samples. Only sex and age were shared with the researchers along with a donor ID number. The ages of the female donors were 22, 23, 25, 26, 36, and 65; the ages of the male donors were 24, 27, 29, 31, 33, and 63. The mean ages for female and male subjects were 32.8 and 34.5 yrs (*p* = 0.86). After collection, the samples were shipped by FedEx overnight to RINSC and CINF. Samples were shipped to CINF despite its being closely located to the medical center, so that blood handling would be as similar as possible between the two sites. Package temperature was monitored during shipping using a USB temperature logger.

### Radiation dosimetry and exposure

Irradiation was performed on two days, roughly a week apart, with 6 donors per day.

At each site, 5 samples of blood from each donor were exposed to 5 different radiation doses, with one sample per dose: 0, 0.5, 1, 2, and 4 Gy.

*At RINSC*: The thermal column of the nuclear reactor was used for irradiation with reactor power of approximately 1.8 Megawatts yielding a flux of approximately 10^8^ neutrons/cm^2^/second at the position of the samples during irradiation. The dose rate was 1.9 Gy per hour of photons and 2.6 Gy per hour of neutrons, making the output 42% photons and 58% neutrons. The dose rate for photons was measured using a Ludlum model 78 stretch scope survey meter. EBT3 Radiochromic film (Ashland Specialty Chemicals, Wayne NJ) was calibrated using the CINF neutron field (see below) correlating optical density and total dose. We then matched optical density between films irradiated at RARAF and RINSC, as a surrogate for total dose, using this to determine the total dose rate for neutrons and photons, subtracting the photon dose rate to determine neutron dose rate and correcting for the approximately 80% neutron dose at CINF adjusted by a measured photon calibration curve. Blood tubes were tilted during irradiation.

*At CINF*: The neutron field is generated using a mixed beam of 5 MeV protons and deuterons impinging on a thick beryllium target (Xu et al. 2015). In this experiment a total beam current of 9 µA was used, resulting in a neutron dose rate of approximately 1 Gy/h with an additional 0.24 Gy/h (19% of total dose) of gamma rays. Dosimetry was performed as described previously (Xu et al. 2015), using a tissue equivalent proportional chamber to measure total dose and a GM counter to measure the photon component.

To match the RINSC neutron/photon ratio, samples were irradiated as shown in Table 1, with the X ray boost dose delivered using a RadSource RS2000 (160 kVp, 25 mA, shelf position 1; Custom Thoraeus filter (0.8 mm Sn/0.3 mm Cu/1 mm Al); HVL 3 mm Cu; dose rate 0.16 Gy/min). The X-ray doses were delivered as soon as possible following the neutron doses, with about 5 min between the end of neutron exposure and the start of the X-ray boost exposure.

**Table 1:** Addition of x-ray dose boost for samples irradiated at CINF

Nominal dose	Neutron dose	Concomitant gamma dose	X ray boost dose
0.5 Gy	0.29 Gy	0.07 Gy	0.14 Gy
1 Gy	0.58 Gy	0.14 Gy	0.28 Gy
2 Gy	1.16 Gy	0.28 Gy	0.56 Gy
4 Gy	2.32 Gy	0.56 Gy	1.16 Gy

As the Ferris wheel has 18 positions and 24 tubes needed to be irradiated, six blood tubes per dose (initially only 0.5 and 1 Gy) were placed horizontally in holders on a Ferris wheel rotating around the target at 0.5 RPM. Halfway through the prescribed dose, tubes were flipped end to end to provide isotropic exposure as described previously (Garty et al. 2017). When the 0.5 Gy tubes had received the full dose, they were removed and replaced with the 4 Gy tubes. When the 1 Gy tubes were done, they were removed, irradiation continued until the 4 Gy tubes needed to be flipped and then the 2 Gy tubes loaded. Two water-containing tubes were placed on either side of the blood containing tubes to maintain similar scatter dose. This irradiation scheme is illustrated in a figure in the Supplementary Information.

At both sites following irradiation, an equal volume (2.7 mL) of RPMI medium (RPMI1640 + 10% heat-inactivated FBS + 1x penicillin/streptomycin; Thermofisher, Waltham, MA) was then added to each sample, and the samples were incubated at 95% humidity, 5% CO_2_, and 37° Celsius for 24 h. This time point was chosen because previous studies in ex vivo exposed blood have shown very similar responses between 6 and 48 h (Paul et al. 2013), and the majority of subsequent ex vivo biodosimetry studies have used the 24 h point. After 24 h, each sample was well mixed with 14.9 mL of PAXgene additive (Qiagen, Germantown, MD), which is double the usual amount per sample, due to the medium doubling the volume. This resulted in two PAXgene tubes per sample. Samples were then frozen at -20° Celsius (for approximately a month) until being shipped to the company Azenta Life Sciences (formerly Genewiz) for RNA sequencing. Samples from donors 1–6 were irradiated and processed on a different day from samples from donors 7–12.

### RNA sequencing

RNA extraction of one set of PAXgene tubes and sequencing were done by Azenta, at their laboratory in South Plainfield, NJ. Library preparation with rRNA depletion was performed with the QIAGEN Fast Select rRNA HMR and Globin kit and the NEBNext Ultra II RNA Library Preparation kit. Fragmentation of enriched RNA was performed at 94 °C over 15 min. cDNA was produced, with steps of index addition and library enrichment including PCR. Samples were sequenced on an Illumina HiSeq, at 2 by 150 base pairs. For 3 of the 120 samples, extraction was repeated using the second tube, due to the initial sample yielding RIN < 6, with the second extraction used in the experiment: CINF 1 Gy female age 26 (sample ID 5), CINF 4 Gy female age 26 (sample ID 9), and CINF 2 Gy male age 63 (sample ID 27). The data are available in the Gene Expression Omnibus under accession number GSE308422.

### Differential expression analysis

Quality check of the data was completed using FastQC (Andrews 2010). The consistently high-quality base scores observed in the FastQC analysis justified the retention of the raw reads for further analysis. The reads were successfully aligned to the GRCh38 reference genome using HISAT2 (Pertea et al. 2016; Kim et al. 2015, 2019), with an overall alignment rate of 91.18%. Gene expression levels were quantified from the RNA-seq data using FeatureCounts (Liao et al. 2014) to create a gene count matrix. Normalization and differential expression analyses were completed using the Bioconductor package DESeq2 in R (Huber et al. 2015; Love et al. 2014; Team 2019). In DESeq2, the model used was source+dose and the adjusted p value cutoff used was 0.05. Shrinkage correction was applied but was found to cause the vast majority of genes to shrink to near zero when analyzing donors 1–12 together, as seen in MA plots. A shrinkage correction was therefore omitted from the final generation of differentially expressed gene lists. The main DESeq2 code is included in the Supplementary Information. During initial analysis, most samples from donors 10, 11, and 12 were found to be anomalous outliers, so all samples from these three donors have been excluded from subsequent analysis. Results from these donors are available in the Supplementary Information.

Venn diagrams were made using the Venny 2.0 Venn diagram software at https://bioinfogp.cnb.csic.es/tools/venny/. Heatmaps were made using the R (CoreTeam 2015) package pheatmap (Kolde 2025), following the vignette (Love et al. 2025) for deseq2 (Love et al. 2014). Figures 2b and c were made using Prism Graph.

### Analysis of overrepresented gene sets

In the analysis of gene sets, DAVID (Dennis et al. 2003) was used to identify gene sets that were overrepresented. Differentially expressed genes from the DESeq2 analysis were used, and the cutoff for significance was a Benjamini corrected p value of 0.05. The internal database for DAVID uses gene sets from several repositories, including KEGG (Kanehisa and Goto 2000), UniProt (UniProt 2023), Gene Ontology (Ashburner et al. 2000; Gene Ontology et al. 2023), Interpro (Paysan-Lafosse et al. 2023), Smart (Letunic and Bork 2018; Letunic et al. 2021), and Biocarta (Rouillard et al. 2016).

### Dose correlation analysis

The gene count matrix was also loaded into BRB-ArrayTools v.4.6.2 (Simon et al. 2007) where, after DESeq2 normalization, quantitative trait analysis was performed to obtain lists of genes from each site with a significant (*p* < 0.001; FDR < 5%) Spearman correlation with dose. The genes found to have a statistically significant correlation were then used with DAVID (Dennis et al. 2003) to find gene sets that were overrepresented among the dose-correlated genes. Relative fold-change of differentially expressed genes was also calculated in BRB-ArrayTools using the class comparison function for plotting of dose response curves.

### Multidimensional scaling (MDS) analysis

Multidimensional scaling analysis was run using BRB-ArrayTools v.4.6.2 (Simon et al. 2007). Results of the analysis were coordinates for each sample, along 3 axes. The samples were plotted using the “scatter3” command in MATLAB. The genes used in the MDS analysis were those found to be significantly correlated with dose in the CINF samples by the dose correlation analysis described above.

## Results

**Fig. 1:**
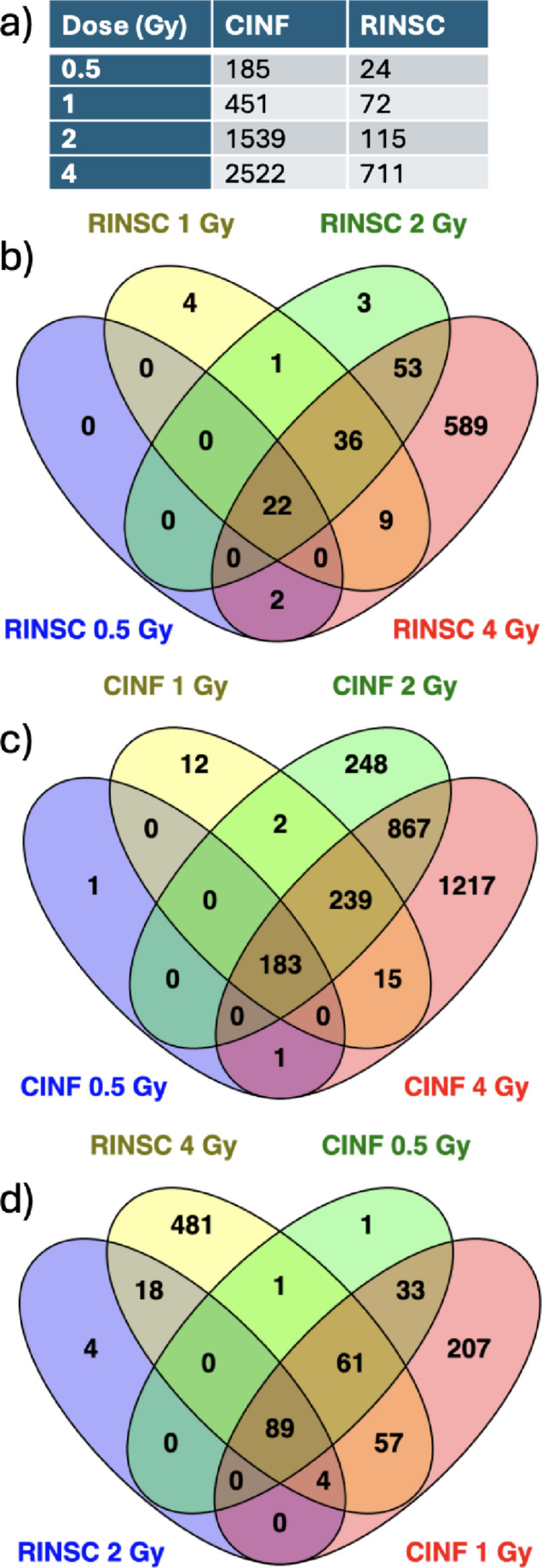
Summary of differential expression results and overlap of gene sets at each dose. (**a**) table with differentially expressed gene counts at each dose (0.5 Gy, 1 Gy, 2 Gy, 4 Gy) for both experimental sites (CINF and RINSC). (**b**) Venn diagram of overlap between all four doses at RINSC. (**c**) Venn diagram of overlap between all four doses at CINF. (**d**) overlap between highest doses at RINSC (2 Gy and 4 Gy) and lowest doses at CINF (0.5 Gy and 1 Gy)

The results of the differential expression analysis are summarized in Fig. 1a. Full lists of differentially expressed genes are available in the Supplementary Information. In the CINF samples, the number of differentially expressed genes increases from 185 at dose 0.5 Gy, to 2522 at dose 4 Gy. In the RINSC samples, the number of differentially expressed genes increases from 24 genes at 0.5 Gy to 711 genes at 4 Gy. Figure 1b shows the overlap between different doses at RINSC, where there were 22 genes that were differentially expressed in all four doses. Figure 1c shows the overlap between different doses at CINF, where 183 genes were differentially expressed in all four doses. Figure 1d shows the overlap in differentially expressed genes between doses 0.5 Gy and 1 Gy at CINF and doses 2 Gy and 4 Gy at RINSC. The differentially expressed genes from the CINF 1 Gy and RINSC 4 Gy exposures have an overlap of 211 genes, while overall there are 89 differentially expressed genes in common between the two high dose RINSC exposures and the two lower dose CINF exposures.

**Fig. 2:**
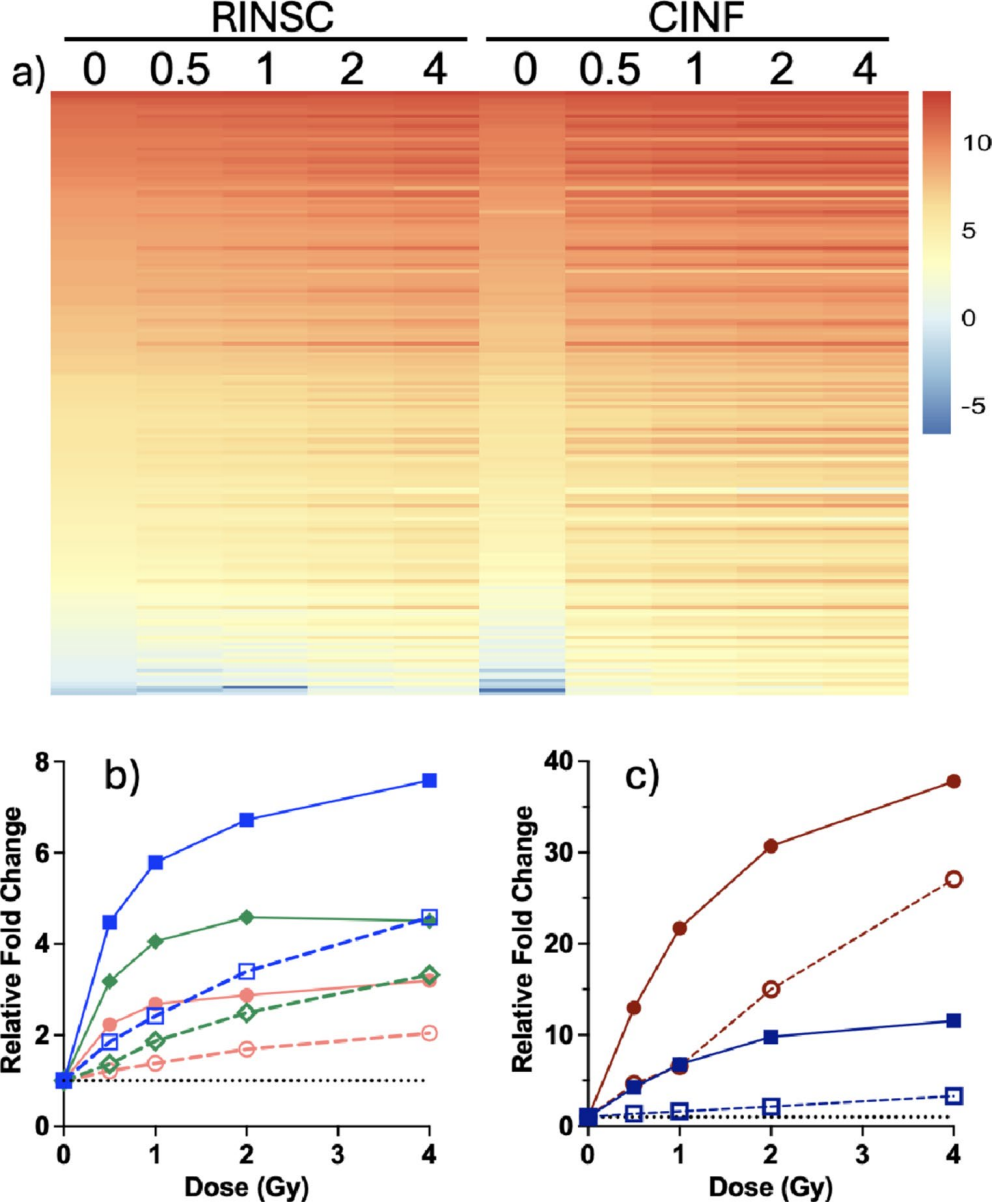
Gene expression after irradiation at the two sites. **a**) Heat map of log2 average expression for every experimental group, for the 183 genes found to be differentially expressed in every dose at CINF. The general pattern of differential expression is very similar between CINF and RINSC, despite the generally higher fold change values in the CINF samples. The value 0.01 was added to each average expression value before the log was taken, to allow for the log to be taken for any zero values. Values plotted here are available in the Supplementary Information. **b**) Dose response of common radiation responsive genes at CINF (solid lines and symbols) and RINSC (open symbols and dashed lines) calculated from RNA-Seq data. Genes are DDB2 (blue), ZMAT3 (green), XPC (pink). Dotted line represents the level in controls. **c**) Relative fold change of PHLDA3 (red) and TNFSF4 (dark blue) at CINF (solid lines and symbols) and RINSC (open symbols and dashed lines). Dotted line represents the level in controls

In order to compare the trends of gene expression changes at the two sites, independent of the level of significance of individual doses, we constructed a heatmap of the average expression in each sample group for the 183 genes that were differentially expressed in all four doses in the CINF samples (Fig. 2a). Notably, the general pattern of expression is very similar in all CINF and RINSC samples, even though the values of the relative expression values are lower in the RINSC samples. The lower values of expression in the samples irradiated at RINSC could be explained by the different energy of neutrons at the RINSC irradiation source and their associated lower values of equivalent dose (ICRP 2007). To further illustrate the patterns of response seen at the two sites, the relative fold change of several typical radiation responsive genes from Fig. 2a have been plotted as dose responses at the two sites (Fig. 2b and c). There is a trend of increasing response with increasing dose for all genes at both sites, but with a larger magnitude of response in the CINF samples.

**Fig. 3:**
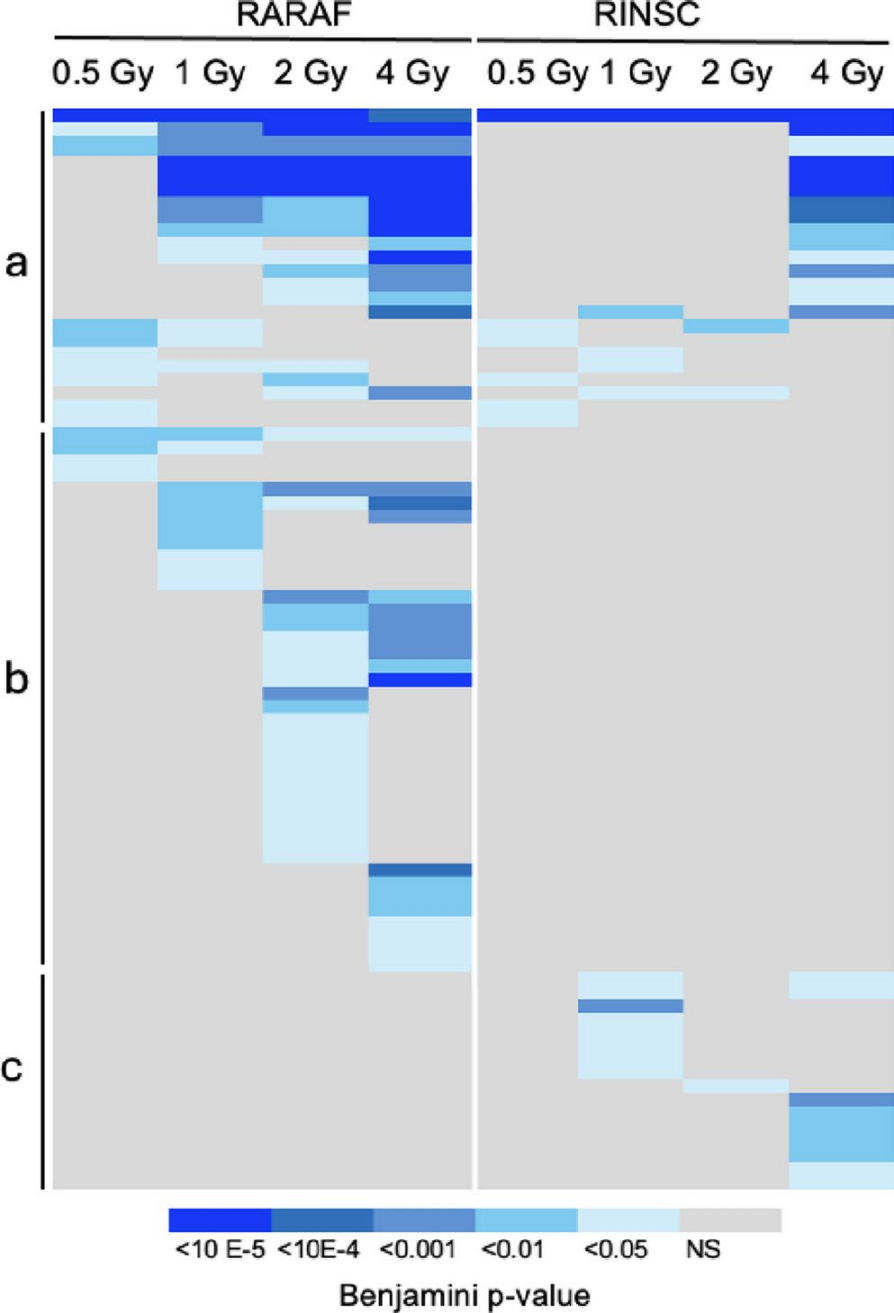
Gene ontology analysis using DAVID. (**a**) Biological processes and pathways with Benjamini *p* < 0.05 are illustrated for all exposures and are color coded according to the scale bar. Only the p53 signaling pathway was significantly over-represented among genes responding to all exposures. Many processes and pathways typically affected by radiation exposure were significantly enriched among genes responding to some doses at both sites, including functions related to DNA damage response, telomere organization, chromatin, transcription, immune response, and apoptosis (Region a). Processes significantly enriched only at CINF included additional DNA damage, chromatin, cell death, transcription, adhesion, immune, and signaling functions (**b**), while further functions related to signaling, cell death, proliferation, and immune functions were enriched only at RINSC (**c**). Full annotation of pathways and processes and their associated Benjamini p values is available in the Supplementary Information File

The significantly differentially expressed genes were assessed for over-represented biological processes and pathways (including KEGG, SMART, BioCarta, and others) using DAVID analysis (Dennis et al. 2003). The results are summarized in Fig. 3, and full results are available in the Supplementary Information. All exposures resulted in significant enrichment of the p53 signaling pathway. Additional processes typically seen after irradiation, including apoptosis, DNA damage response, chromatin, immune response, transcription, and cancer were also overrepresented at both sites. At CINF only, additional overrepresented gene sets related to cancer, chromatin, cell death, adhesion and TNF were seen at various doses, with immune processes and signaling dominating the higher dose (2 Gy and 4 Gy) response. Additional functions related to cytokines, cell death, and signal transduction were overrepresented only at RINSC after doses of 1 Gy and above.

As the differentially expressed genes shared between all doses in the CINF exposures showed a similar expression pattern across the RINSC doses (Fig. 2a), it was possible that additional genes were affected in the RINSC samples, but not strongly enough to reach significance in the analysis of individual doses. If such genes respond with a dose-dependent trend, they may nonetheless reach significance when considered as a correlation across multiple doses, and this could provide an indication of a true radiation response. We next tested for genes with a significant correlation between expression level and dose at the two sites. This analysis identified 444 genes with a significant (*p* < 0.001; FDR < 5%) Spearman correlation among samples irradiated at CINF, and 107 at RINSC (Supplementary Information). Of these genes, 94 were common to both sites.

The dose-correlated genes were then analyzed by DAVID (Dennis et al. 2003), and the results are shown in Table 2. Biological processes and KEGG pathways overrepresented among the dose-correlated genes at both sites were related to p53 signaling, cytokines, apoptosis, and immune response. Additional apoptosis, immune/inflammatory, and signaling related processes were overrepresented only among the CINF dose-correlated genes. Pathways related to specific cancers, signaling pathways, and DNA damage response were overrepresented only among the RINSC dose-correlated genes.

**Table 2:** DAVID Gene Ontology analysis of dose-correlated genes. Significantly over-represented biological processes or pathways (Benjamini-Hochberg corrected p-value ≤ 0.05) are listed with their adjusted p-values. N.S. indicates not significant

Term (Biological Process or KEGG Pathway)	CINF	RINSC
p53 signaling pathway	6.72 × 10^− 5^	2.01 × 10^− 10^
Cytokine-cytokine receptor interaction	0.000551	0.00163
Apoptotic process	0.00032	0.00905
Immune response	0.000881	0.0186
DNA damage response, signal transduction by p53 class mediator	N.S.^a^	4.28 × 10^− 4^
Chronic myeloid leukemia	N.S.	0.00459
Platinum drug resistance	N.S.	0.00459
Thyroid cancer	N.S.	0.00459
Intrinsic apoptotic signaling pathway in response to DNA damage by p53 class mediator	N.S.	0.00622
Colorectal cancer	N.S.	0.0068
Endometrial cancer	N.S.	0.0131
Human cytomegalovirus infection	N.S.	0.0131
Basal cell carcinoma	N.S.	0.0135
DNA damage response	N.S.	0.0158
Cellular response to UV	N.S.	0.0186
Melanoma	N.S.	0.0212
Positive regulation of apoptotic process	3.4 × 10^− 5^	N.S.
Regulation of cell migration	0.00268	N.S.
Signal transduction	0.00268	N.S.
Positive regulation of gene expression	0.00718	N.S.
Inflammatory response	0.0078	N.S.
Graft-versus-host disease	0.00903	N.S.
Antigen processing and presentation	0.0106	N.S.
ECM-receptor interaction	0.0174	N.S.
Apoptosis	0.0232	N.S.
Cell-cell signaling	0.037	N.S.

## Discussion

The main goal of this study was to assess the degree of similarity in gene expression responses between an irradiator designed to match the neutron energy spectrum at a survivable distance from an IND (CINF) and a nuclear reactor used for research (RINSC), which produces mainly thermal neutrons. With increasing awareness of the importance of the neutron component of dose when developing countermeasures against nuclear events, the use of more widely available research reactors could accelerate progress in this area. The very different neutron energies of these two sources could affect the biological response, however. We found substantially different differential gene expression results from matched doses with the two sources. Broadly, although many of the same genes responded at both sites, the expression changes in the RINSC samples were of generally lower magnitude than those in the CINF samples. Adding the results from dose correlation and gene ontology analyses further supported a broad similarity of the underlying response at the two sites, with the main difference being the level of response per given dose. This supports the potential usefulness of research reactors for biodosimetry studies in the future. Figure 1d compares lower doses at CINF with higher doses at RINSC. This is because the RINSC source produces primarily thermal neutrons whereas CINF produces primarily fast neutrons, which are expected to produce a greater biological effect per Gy. The difference in biological effect from different energy neutrons can be quantified using the radiation protection concept of equivalent dose (ICRP 2007). Using Eq. 4.2 from ICRP report 103 (ICRP 2007), the neutron energy spectrum of the CINF source (Xu et al. 2015), the neutron energy of thermal neutrons, and the 58% to 42% neutron to photon content for both sources, the equivalent dose (in Sieverts) is 5.3 times larger for the CINF samples than for the RINSC samples. Further details of this calculation are available in the Supplementary Information. Although the equivalent dose is not based on the gene expression endpoints measured here, the expected difference in biological effect suggests it may be useful to compare the results of a lower dose at CINF to a higher dose at RINSC.

A common way of comparing radiations of different qualities is using the Relative Biological Effectiveness (RBE), calculating ratio of the dose of a reference radiation (generally 1 Gy/min x rays) and the dose of the other radiation of interest required that is required to obtain the same response at a given dose. RBE is known to vary both by dose and by the outcome variable being tested. RBE of the CINF neutron field for gene expression previously showed an average RBE for gene expression of 1.8, but for individual gene-dose combinations, RBE ranged from less than one to 3.9 (Broustas et al. 2017a). While a similar study of RBE at RINSC may be of interest in the future, we focused here on a direct comparison of the effects of the two sources. It should also be noted that the two neutron sources produced exposures of different dose rates, around 4.5 Gy per hour at RINSC and 1 Gy per hour at CINF.

Dose rate effects can play an important role in biological responses, including gene expression, and while there generally appears to be little or no dose rate effect with high LET, the x-ray component of the exposures also differed (4.3 cGy per minute at RINSC and an average of 0.3 cGy per minute at CINF) and some effects on gene expression have been noted within this range (Amundson et al. 2003; Ghandhi et al. 2015). It is not known how these potential differences may interact with the neutron damage component, but this should be taken into consideration in interpreting these results, and may be of interest to explore experimentally in future studies.

The over-representation of some gene ontology categories only at one site or in limited doses should be interpreted cautiously, as this does not necessarily indicate a unique biological response, but rather the analysis helps provide a general overview of affected processes. For instance, one of the categories enriched only among differentially expressed gene sets from the RINSC samples (Fig. 3 section c) is the KEGG pathway “hsa04210:Apoptosis”, which appears only significantly enriched among the 1 Gy RINSC genes. This does not mean that apoptotic processes were only engaged in this one sample; indeed, several gene ontology biological processes related to apoptosis (GO:0043065 ~ positive regulation of apoptotic process, GO:0042771 ~ intrinsic apoptotic signaling pathway in response to DNA damage by p53 class mediator, and GO:0006915 ~ apoptotic process) appear in region “a” of Fig. 3 and were seen to be over-represented in gene sets from both sites. Looking more closely at the KEGG Apoptosis pathway, we see the RINSC sample where this was significant includes 6 genes annotated with this pathway, out of 38 genes from this sample with annotations. Overall, this results in a 9.9-fold enrichment of this category in the RINSC gene list compared with the abundance of this annotation across the genome. In contrast, differentially expressed genes from CINF included up to 17 genes with the KEGG apoptosis annotation, but in this case the gene list had a total of 547 annotated genes, resulting in an enrichment of only 1.95 fold. In this case, the uncorrected p value was 0.013, but with multiple comparison correction the Benjamini p value rose to a non-significant 0.15. Generally, the DAVID gene ontology analysis of the differentially expressed genes shown in Table 2 supports an overall similarity between the biological responses to the neutron exposures given at the two sites. This was not evident from simply comparing the numbers of differentially expressed genes from each dose and site.

While the findings presented here do support the possibility of using research reactors for countermeasures research, many experiments are still needed to enable practical use. One approach for biodososimetry is development of a signature of dose-correlated genes and an algorithm for dose reconstruction followed by validation using qRT-PCR in test cohorts. We have developed dose reconstruction for x rays using gene expression, but have not yet optimized the approach for neutron exposures. (Ghandhi et al. 2019, 2022; Broustas et al. 2023). Such signature optimization can also help compensate for the often-seen high dose flattening of gene expression responses, which is seen here with the CINF exposures (Fig. 2b and c), both by selecting the most highly dose-correlated genes and by incorporating both up and down regulated genes (Ghandhi et al. 2022).

Another approach, perhaps more directly relevant, is to develop signatures that predict biological outcome, such as the degree of hematological acute radiation syndrome (Port et al. 2017; Blakely et al. 2018). It is ultimately the injury we wish to treat, and eliminating the surrogate of dose also eliminates the need to reconstruct details of the exposure such as neutron percentage or dose rate. While non-human primates models are the most relevant to humans, these have their own limitations, and much can also be learned from rodent models (Rogers et al. 2020; Pannkuk et al. 2025), which would be possible to incorporate into future studies extending the work described here.

Although this study used both male and female donors, it is not powered to detect small sex differences in radiation response, and we did not analyze the sexes separately, focusing rather on common responses. We have previously tested sex as a variable for dose reconstruction models, but it has not emerged as a significant contributor to variation (Ghandhi et al. 2022; Shuryak et al. 2023). However, a better understanding of the limitations and appropriate application of radiation biodosimetry using gene expression will nonetheless require broad demographic studies to test potential confounding factors and incorporate them into dose reconstruction algorithms as needed. Large studies testing variables such as sex, age, and race are underway with x-rays, and would be interesting to extend to gene expression reconstruction of neutron dose. Pre-existing conditions or medication use, particularly those that result in immunosuppression or immunocompromise or that affect inflammation status (viral infections, trauma, diabetes, NSAIDS) may also be of importance, as these share some response pathways with radiation.

The present study examined responses only at a 24 h timepoint. Previous studies in ex vivo exposed blood have shown very similar responses between 6 and 48 h (Paul et al. 2013), and most of our ex vivo biodosimetry work has focused on the 24 h timepoint. Gene expression is known to be highly dynamic, however, and the temporal dynamics can vary with both the dose and dose rate of exposure (Amundson et al. 1999, 2003). It would be interesting to examine the temporal dynamics of gene expression following neutron exposures of different energies and dose rates in the future, or to extend this work to animal models to characterize responses across longer times post exposure.

Overall, our findings suggest that neutron sources cannot be used interchangeably, but that the energy spectra most relevant to a particular scientific question should be taken into consideration. Thus, while a specialized facility such as CINF may be most appropriate for countermeasure testing and emergency biodosimetry development, it may be possible to generate meaningful data for such studies using research reactors. Additional experiments will be needed, however, to better understand the relationships between neutron energy, dose or injury level, and gene expression or other endpoints of interest.

## Supplementary Information

Below is the link to the electronic supplementary material.

Supplementary Material 1

## Data Availability

Raw and normalized gene expression data will be published on the Gene Expression Omnibus (accession number GSE308422).
